# Clone-Dependent Expression of Esca Disease Revealed by Leaf Metabolite Analysis

**DOI:** 10.3389/fpls.2018.01960

**Published:** 2019-01-09

**Authors:** Florian Moret, Christelle Lemaître-Guillier, Claire Grosjean, Gilles Clément, Christian Coelho, Jonathan Negrel, Lucile Jacquens, Guillaume Morvan, Grégory Mouille, Sophie Trouvelot, Florence Fontaine, Marielle Adrian

**Affiliations:** ^1^Agroécologie, AgroSup Dijon, Centre National de la Recherche Scientifique, Institut National de la Recherche Agronomique, Université de Bourgogne, Université de Bourgogne Franche-Comté, Dijon, France; ^2^Chambre Régionale d’Agriculture de Bourgogne Franche-Comté, Bretenière, France; ^3^Institut Jean-Pierre Bourgin, Institut National de la Recherche Agronomique, AgroParisTech, Centre National de la Recherche Scientifique, Université Paris-Saclay, Versailles, France; ^4^UMR PAM Université de Bourgogne/AgroSupDijon, Institut Universitaire de la Vigne et du Vin Jules Guyot, Dijon, France; ^5^Chambre d’Agriculture de l’Yonne, Auxerre, France; ^6^SFR Condorcet CNRS 3417, Université de Reims Champagne-Ardenne, Unité Résistance Induite et Bioprotection des Plantes, Reims, France

**Keywords:** *Vitis vinifera*, grapevine trunk diseases, clone, metabolomics, Esca, 3D fluorescence

## Abstract

Grapevine trutk diseases, especially Esca, are of major concern since they gradually alter vineyards worldwide and cause heavy economic losses. The expression of Esca disease symptoms depends on several factors, including the grapevine cultivar. In this context, a possible clone-dependent expression of the Esca disease was studied. Two clones of ‘Chardonnay’ grown in the same plot were compared according to their developmental and physiological traits, metabolome, and foliar symptom expression. Analysis of their leaf metabolome highlighted differences related to symptom expression. Interestingly, the content of a few specific metabolites exhibited opposite variations in leaves of symptomatic shoots of clones 76 and 95. Altogether this study showed a clone-dependent expression of Esca disease in ‘Chardonnay’ and the relevance of GC-MS and 3D fluorescence methods to analyze the impact of the disease on the leaf metabolome.

## Introduction

Grapevine trunk diseases (GTDs) gradually alter vineyards worldwide, causing important economic losses (De la Fuente et al., 2016). The subsequent replacement of dead grapevines is indeed estimated superior to 1.5 billion dollars per year (Hofstetter et al., 2012). In California, yield losses in many vineyards over age 10 can reach over 90% (Baumgartner et al., 2014). In France, close to 13% of the vineyard is now affected (Bruez et al., 2013). In the past, the impact of such diseases, especially Esca, was limited thanks to the use of sodium arsenite. In 2003, because of its high toxicity on human health and environment (Spinosi et al., 2009), this fungicide was prohibited in Europe. No efficient treatment is since available to control GTDs (Spinosi et al., 2009; Gramaje et al., 2018; Mondello et al., 2018). Research is therefore required to gain a better knowledge of these diseases and their expression in order to limit their development.

Esca disease, Botryosphaeria dieback and Eutypa dieback are the most relevant GTDs. Esca complex includes several syndromes (Surico et al., 2008; Surico, 2009; Mondello et al., 2018) among which « grapevine leaf stripe disease ». The main causal agents are the ascomycetes *Phaeomoniella chlamydospora* and *Phaeoacremonium minimum*, and the basidiomycete *Fomitiporia mediterranea*. These pathogens are found in the woody tissues of perennial organs and, to a lesser extent, in annual canes, but never in leaves where symptoms express (Larignon and Dubos, 1997; Graniti et al., 2000; Fourie and Halleen, 2002; Retief et al., 2006). Their development leads to two possible forms of disease expression in leaves: a chronic form characterized by the occurrence of typical tiger-like necrosis and chlorosis, and an apoplectic form, with a sudden leaf wilting followed by the rapid death of one part up to the whole plant (Mugnai et al., 1999; Fontaine et al., 2016).

Several factors could increase the grapevine susceptibility to GTDs, mainly climate, vine age, soil fertilization, rootstock and cultivar (Graniti et al., 2000; Surico et al., 2004, 2006). Presently, no tolerant cultivars could be identified and the level of disease expression of one cultivar can vary with region and year. One previous study compared clone response to Esca disease, and reported visual differences in symptom expression between ‘Sauvignon’ clones, but not for ‘Chardonnay’ clones (Murolo and Romanazzi, 2014). In this context, the purpose of the present study was to use several approaches to highlight a clone-dependent Esca disease expression. Two clones (76 and 95) of the ‘Chardonnay’ cultivar grown in a same plot were therefore compared according to their developmental and physiological traits, metabolome, and Esca foliar symptom expression.

## Materials and Methods

### Experimental Plot

Experiments were conducted in 2015 in a plot located in Chablis vineyard (126 m above sea level, Burgundy, France; GPS coord.: 47°47′46.96″N, 3°47′26.17″E). This plot is characterized by a clayey soil, an alkaline pH (8.3), and a high Cationic Exchange Capacity (CEC) of 247.7 meq/kg. It was longitudinally divided into two parts and planted in 2002 with the two ‘Chardonnay’ clones 76 and 95 grafted on Fercal rootstock. Vines are trained in a double Guyot at a density of 1 m × 1.50 m, and fertilized once a year with organic Vegethumus^TM^ (1t/ha) (Frayssinet, Rouairoux, France), organic farming being used as protection strategy.

For each clone, two series of 10 successive vines (i.e., 20 vines) were selected in the plot to follow agronomic traits, and for berry sampling (see below). Esca foliar symptoms were visually observed at the end of August, between veraison and maturity stages on the whole plot vines (i.e., 2201 and 1974 vines, for clone 76 and 95, respectively). Notation was made using the following classes: apoplectic, chronic, or unproductive vines (i.e., dead, missing, or replanted vines).

### Climate Data

Temperature (°C) and precipitation (mm) data over the 2005–2015 period were provided by a meteorological station (Cimmel Enerco 404) located close to the plot, and representative for the climate conditions of the experimental plot.

### Agronomic Traits

The percentage of bud burst, fertility and the vigor were determined in August on 20 vines per clone. They were calculated as follows:

Bud burst percentage = (number of shoots/number of buds remaining after Chablis pruning) × 100

Fertility = number of clusters/number of shoots

Vine vigor was determined by measurements of cane diameter using calipers. Measurements were performed between the first and second node of the third cane starting from the old part of the trunk (Chablis pruning).

### Water Status

Water status of vines was estimated by measurements of the delta ^13^C at the end of August, 10 days before harvest. Analyses were performed on must obtained from 150 berries collected on clusters of the two series of each clone. The samples were analyzed at GISMO Platform (University of Burgundy, France).

### Metabolite Analyses

#### Sampling

**FIGURE 1:**
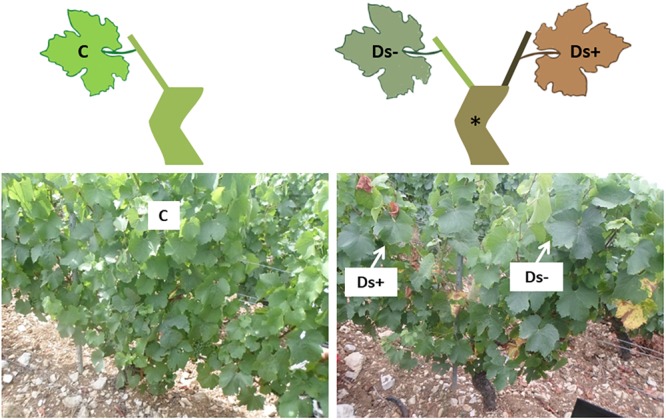
Sampling scheme and pictures. For each clone, leaves were collected from healthy (without visual symptoms, as control, annotated C; left side of the figure) and Esca-diseased vines (^∗^). For Esca-diseased vines, leaf samples were collected from both asymptomatic and symptomatic shoots of the same vines, respectively, annotated Ds– and Ds+ (right side of the figure).

For each clone, leaves (located in the face of bunches) were collected throughout the plot from five healthy (without visual foliar symptoms, as control, annotated C) and five Esca-diseased vines showing partial apoplexy symptoms (annotated D). For each healthy vine, two leaves were collected and pooled (10 leaves collected in total). For Esca-diseased vines, 10 leaves were collected from both symptomatic (three leaves in total) and asymptomatic shoots (seven leaves in total) of the same vines, and respectively, annotated Ds+ and Ds- (Figure 1). Ds+ leaves were still green without apparent necrosis. Sampling was performed at the end of July, just at the beginning of symptom appearance. Samples were immediately frozen in liquid nitrogen and stored at -80°C until use. They were ground into a fine powder in liquid nitrogen before use.

#### Leaf Photosynthetic Pigments

Pigments were extracted from leaf powder (30 ± 5 mg) with 5 mL of 80% (v/v) acetone supplemented with 0.5% (w/v) CaCO_3_ overnight at 4°C under continuous agitation. After centrifugation (10,000 × *g* for 10 min at 4°C), the supernatant was collected and absorbance was measured at 470, 647, and 663 nm. Pigment concentrations were then calculated according to Lichtenthaler method (Lichtenthaler, 1987).

#### GC-MS Analysis

Samples preparation, analysis, and data processing were performed as previously described (Fiehn et al., 2006, 2008; Krzyzaniak et al., 2018). Briefly, leaf samples (50 ± 10 mg) were resuspended in 1 mL of frozen water:acetonitrile:isopropanol (2:3:3) containing ribitol at 4 μg/mL and extracted for 10 min at 4°C with shaking at 1,400 rpm. After centrifugation (20,000 × *g*, 5 min), 100 μl supernatant were collected and dried for 5 h in a SpeedVac vacuum centrifuge. Samples were TMS-derivatized and analyzed using an Agilent 7890B gas chromatograph coupled to an Agilent 5977B mass spectrometer. For processing, data files were converted in NetCDF format and analyzed with AMDIS^1^. A home retention indices/mass spectra library built from the NIST, Golm^2^, and Fiehn databases and standard compounds were used for metabolite identification. Peak areas were also determined with the Targetlynx software (Waters) after conversion of the NetCDF file in Masslynx format. AMDIS, Target Lynx in splitless and split 30 mode data were compiled into a single Excel file for comparison (Supplementary Table S1). After blank mean substraction, peak areas were normalized to ribitol and fresh weight (in μg/mg fresh weight).

#### HPLC Analysis

Some of the collected samples (5 C, 3 Ds- and 2 Ds+ ones of each clone) were prepared by overnight extraction in methanol (0.1 g/mL). Analysis of phenolics was performed by HPLC using a Beckman System Gold chromatography system equipped with a diode array detector Model 168 and a Beckman 507 sample injector equipped with a 20 μL sample loop as described by Krzyzaniak et al. (2018). Phenolics were separated on a Kinetex C18 column (4.6 × 100 mm, 2.6 μm, Phenomenex) at a flow rate of 1 mL/min and a mixture of solvent A (1.5% phosphoric acid in MilliQ water) and solvent B (100% acetonitrile) as mobile phase. Phenolics were eluted within 30 min with a linear gradient from 0 to 40% solvent B. Retention times were 6.43 min for *trans*-caffeoyltartaric acid, 7.58 min for *trans*-coumaroyl-tartaric acid, 11.89, 12.07, and 12.19 min for quercetin glycosides (quercetin-3-*O*-glucoside, rutin, and quercetin-3-*O*-galactoside, respectively), and 13.02 min for kaempferol-3-*O*-glucoside. Identification of the different phenolics was performed by comparison of their retention times and UV-vis spectra with those of reference compounds. Amounts of each phenolic compound occurring in the leaves were averaged from peak areas at 310 nm.

#### 3D Fluorescence Analysis

Qualitative variations of fluorescent compounds were measured in the same set of samples (5 C, 3 Ds- and 2 Ds+ replicates of each clone) analyzed by HPLC. Grounded leaves (200 mg) were mixed with methanol at 0.1 g/mL, centrifugated (3,000 × *g*, 5 min, room temperature), and the supernatant was then diluted in methanol (1:300). Methanolic extracts were analyzed with a Horiba Aqualog Horiba^®^ spectrofluorimeter using a 1-cm pathlength quartz cuvette. Excitation-Emission Matrices (EEMs) were acquired from 600 to 225 nm (3 nm steps for excitation) and from 211 to 617 nm (3.34 nm steps for emission) wavelengths.

Data were mathematically corrected in order to minimize inner filter effects, withdraw Rayleigh scattering and normalized to a Starna 1-ppm quinine sulfate reference cell. PARAFAC modeling was carried out using the drEEM tutorial (Murphy et al., 2013) accompanying Matlab code. The PARAFAC model was built with a number of components sufficient enough to best fit the variability of the EEM dataset and to validate through the core consistency and split half validation procedure. After model validation, *F*_max_ values for each PARAFAC component were obtained in order to represent the original EEM intensities.

### Statistical Analysis

Agronomical parameters (cane diameter, bud burst, fertility ratio) and delta ^13^C data were analyzed with Mann–Whitney tests (*P* < 0.05). Pigment concentrations were analyzed by Kruskal–Wallis test followed by Dunn’s sum-rank pairwise *post hoc* test with Holm correction (*P* < 0.05). Statistical significance is acknowledged between two conditions if they have no letter in common. These analyses were made using R software (R Core Team, 2018).

For statistical analysis of GC-MS data, values were processed with the Perseus^®^ software (version 1.5.1.6^3^) which performed the z-scoring. Multivariate analysis was performed with Pearson correlation (*P* < 0.05). Significant metabolites were determined using two-sided Student *t*-test and permutation-based FDR. S0 constant for variance correction was set at 0 for comparisons and the level of FDR was set at 5%. Subsequent volcano plots (-log_10_ for *t*-test statistical *p*-value vs. differences of means) illustrate significant compounds whose difference changes undergo a FDR < 0.05. Principal component analyses (PCA) were realized with R-Studio software^4^.

## Results

### Weather Conditions

Weather conditions in 2015 are presented in Supplementary Figure S1 and Supplementary Table S2. The average monthly temperature from June (18.8°C) to August (20.7°C) was higher than the ten past year ones, with a maximum of 22.2°C in July. The precipitations were weak in June and July (less than 30 mm per month), and increased to 133 mm in August. The temperature conditions were fairly common when compared to those of the past years, whereas pluviometry was lower than average during the first half of the summer.

### Agronomical Traits

The bud burst percentage of clone 76 (91.52%) was significantly lower than that of clone 95 (95.37%). The plant vigor was not significantly different between clones, with a mean cane diameter of 8.15 ± 1.05 mm and 9.43 ± 3.51 mm for clones 76 and 95, respectively. The fertility ratio of clone 95 was significantly higher than that of clone 76 (values of 1.55 and 1.14, respectively). Values obtained for delta ^13^C measurements in musts were not significantly different between the two clones (-23.56 and -23.45 for clones 76 and 95, respectively).

### Foliar Symptom Expression

**FIGURE 2:**
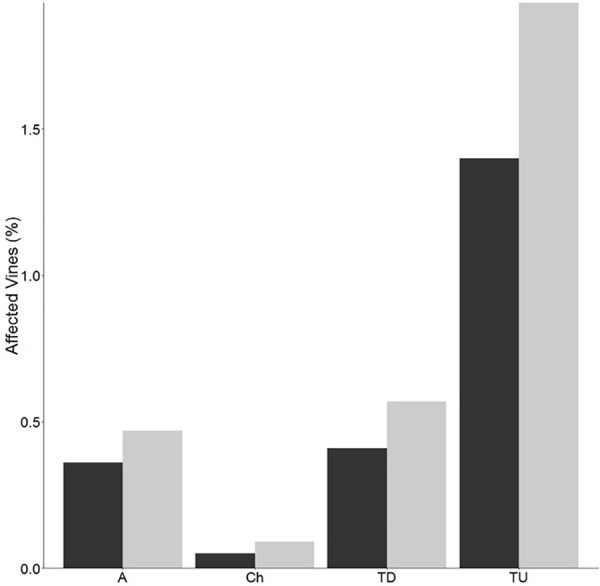
Disease expression. Percentages of apoplectic (A), chronic (Ch), total diseased (TD), and total unproductive (TU) grapevines of clone 76 (black bars) and clone 95 (gray bars) within the plot. Grapevines were counted at the end of August 2015. Total diseased percentage was calculated after addition of apoplectic vines and those affected by the chronic form. Total unproductive percentage corresponds to dead, missing, or replanted vines.

The percentage of vines expressing the apoplectic and chronic forms of Esca disease was low but slightly higher for clone 95, compared to clone 76 (Figure 2). The percentage of unproductive vines was also higher for clone 95, but also low (<2%). No virus associated symptoms were observed.

### Metabolite Analyses

For photosynthetic pigments, the content of total chlorophyll and carotenoids obtained for the different samples is presented in Supplementary Table S3. No significant differences could be observed among samples.

**FIGURE 3:**
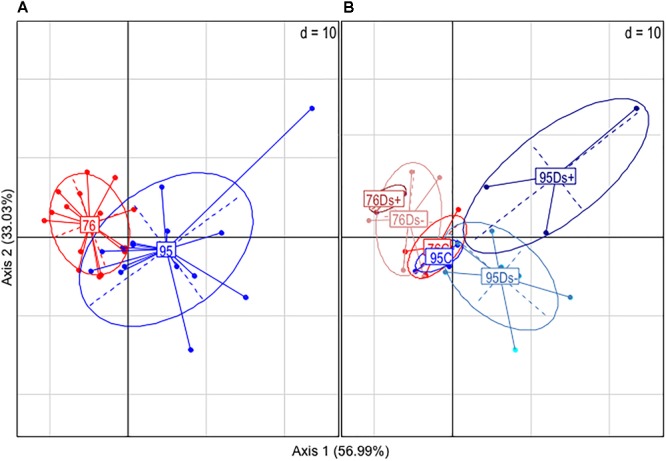
Principal component analysis (PCA) of GC-MS data. Analyses were performed from leaf extracts of ‘Chardonnay’ clones 76 and 95. **(A)** Global analysis of the clone-associated metabolites (177 compounds). **(B)** Detailed PCA illustration of metabolites listed for each clone categories: C (leaf samples from healthy vines), Ds– (leaf samples from asymptomatic shoots of diseased vines), and Ds+ (leaf samples from symptomatic shoots of diseased vines).

Leaf samples were also analyzed by GC-MS for metabolite profiling. A total of 177 compounds was obtained, among which 96 were identified (Supplementary Table S1). Principal component analysis (PCA) allowed partial separation of clone 76 samples from clone 95 ones (Figure 3A). The overlay is due to co-clustering of C samples for both clones (Figure 3B). Ds+ and Ds- sample sets could be distinguished from each other in clone 95 but not in clone 76 (Figure 3B).

**FIGURE 4:**
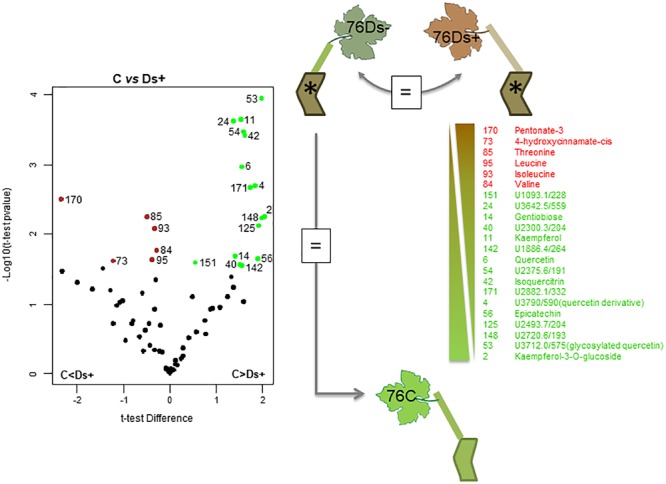
Metabolite variations in leaves of ‘Chardonnay’ clone 76. *T*-test was used to discriminate leaf samples of ‘Chardonnay’ clone 76. The resulting 22 metabolites differently accumulated in Ds+ and C samples are plotted onto a Volcano illustration (left hand panel). Metabolites were considered as significant when undergo a FDR *t*-test difference < 0.05 and a *p*-value < 0.05 (in log scale). Metabolites accumulated in higher amounts in Ds+ samples are plotted in red whereas those accumulated in higher amounts in C ones are plotted in green. Significant metabolites were numbered and listed along a gradient colored scale according to their accumulation in C and Ds+ leaves (right hand panel). No significant metabolites (=) were observed between Ds– vs. Ds+, nor between Ds– vs. C samples.

**FIGURE 5:**
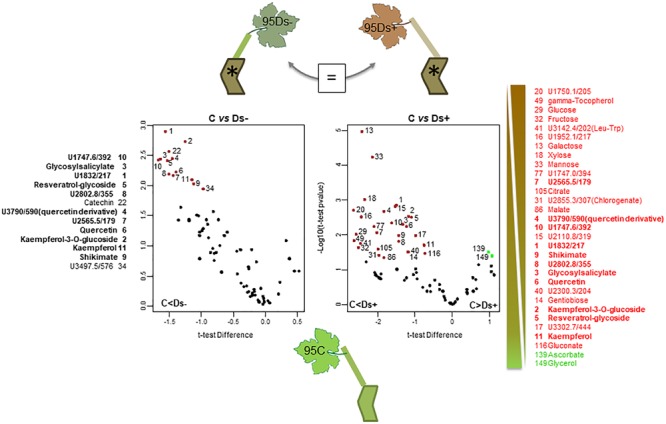
Metabolite variations in leaves of ‘Chardonnay’ clone 95. *T*-test was used to discriminate leaf samples of ‘Chardonnay’ clone 95. The resulting 44 metabolites differently accumulated in C, Ds–, and Ds+ samples are plotted onto Volcano illustrations (center panel). Metabolites were considered as significant when undergo a FDR *t*-test difference < 0.05 and a *p*-value < 0.05 (in log scale). Metabolites accumulated in higher amounts in Ds samples are plotted in red, whereas those accumulated in higher amounts in C samples are plotted in green. Significant metabolites were numbered and listed along a gradient colored scale according to their accumulation in C and Ds leaves (13 between C and Ds–, 31 between C and Ds+). No significant metabolites (=) were observed between Ds– vs. Ds+ samples. Bold characters indicate redundant metabolites between the two significant compound lists.

A subsequent hierarchical clustering analysis allowed a notable discrimination between samples of each clone (Supplementary Figure S2). The significant differences between sample sets were next determined by statistical *t*-test with a permutated FDR < 0.05, and illustrated by Volcano plots when significant. Only identified and putative metabolites were next considered. For clone 76, neither metabolite distinguished Ds+ from Ds- leaves, nor C from Ds- ones (Figure 4). However, differences could be observed between C and Ds+ ones. By comparison to Ds+, C leaves were characterized by a higher content of secondary metabolites, namely kaempferol, kaempferol-3-*O*-glucoside, isoquercitrin, quercetin, a quercetin glucoside and a probable quercetin derivative, epicatechin, and gentiobiose. On the opposite, Ds+ leaves contained higher amounts of an uncharacterized pentonic acid (named pentonate-3), *cis*-4-hydroxycinnamate and the amino acids threonine, leucine, isoleucine, and valine. For clone 95 (Figure 5), metabolite patterns of Ds+ and Ds- leaves were not significantly different. However, C metabolite pattern could be distinguished from those of both Ds+ and Ds- ones. Compared to C leaves, Ds- ones accumulated higher amounts of secondary metabolites including kaempferol, kaempferol-3-*O*-glucoside, quercetin and probable quercetin derivative, resveratrol-glycoside, catechin, shikimate and glycosyl salicylate. When looking at C leaves *versus* Ds+ ones, only the two compounds ascorbate and glycerol were more abundant in C samples while several primary and secondary metabolites were accumulated in Ds+ ones. These primary metabolites were carbohydrates (glucose, fructose, xylose, galactose, mannose, and gentiobiose), together with organic acids (citrate, malate and shikimate). Secondary metabolites were kaempferol, kaempferol-3-*O*-glucoside, quercetin, and putative quercetin derivative, resveratrol-glycoside, glycosyl salicylate, and gamma-tocopherol.

Differences among Ds+ and C samples of clone 76 are due to a rather limited number of compounds (22), and most of discriminant ones (16 over 22) are more abundant in C leaves. For clone 95, more discriminant metabolites (31) were listed; most of them (29) are more abundant in Ds+ leaves. Six common discriminant metabolites, i.e., kaempferol, kaempferol-3-*O*-glucoside, quercetin, quercetin derivative, gentiobiose and an unknown U2300.3/204, were pointed out among Ds+ samples of clones 76 and 95, versus their C equivalent (Table 1). They have opposite variation in both clones: they were indeed found in higher amounts in Ds+ leaves of clone 95 and lower amounts in Ds+ leaves of clone 76.

**Table 1:** Differential accumulation of six discriminant compounds in ‘Chardonnay’ clones 76 and 95.

76Ds+	vs. *#C*	95Ds+
–1.53	Kaempferol	+0.75
–1.56	Quercetin	+1.22
–1.84	U3790/590(quercetin derivative)	+1.83
–2.05	Kaempferol-3-*O*-glucoside	+1.17
–1.41	Gentiobiose	+1.18
–1.52	U2300.3/204	+1.19

*Comparison of GC-MS results obtained for C and Ds+ samples of both clones allowed the identification of six common discriminant compounds. Data listed in 76 Ds+ and 95 Ds+ columns correspond to *t*-test difference values (calculated from Perseus software) obtained for clones 76 and 95, respectively. Symbol + and – indicate over and lower accumulation, respectively, in Ds+ samples vs. C ones. C corresponds to leaf samples of healthy vines, Ds+ to leaf samples of symptomatic shoots of Esca diseased vines.*

**FIGURE 6:**
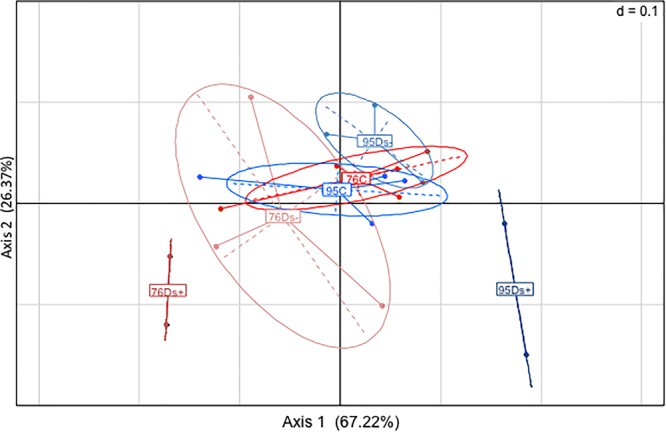
Principal component analysis of HPLC data. PCA plot was built from the quantification of six compounds (*trans*-caffeoyltartaric acid, *trans*-coumaroyltartaric acid, quercetin-glucoside, rutin, quercetin-galactoside and kaempferol-glucoside) in C, Ds–, and Ds+ samples of ‘Chardonnay’ clone 76 (in red) and 95 (in blue). C, leaf samples from healthy vines; Ds–, leaf samples from asymptomatic shoots of diseased vines; Ds+, leaf samples from symptomatic shoots of diseased vines.

HPLC analyses of *trans*-caffeoyltartaric acid, *trans*-coumaroyl-tartaric acid, quercetin-3-*O*-glucoside, quercetin-3-*O*-galactoside, and kaempferol-3-*O*-glucoside were next performed. PCA of HPLC data confirmed the discrimination between Ds+ and C samples of each clone, and Ds+ samples of both clones (Figure 6). These compounds were effectively recovered with a clone-dependent accumulation (Table 2). Thus, *trans*-caffeoyltartaric acid, quercetin-*O*-glucoside, quercetin-*O*-galactoside, and kaempferol-*O*-glucoside were accumulated in Ds+ samples of clone 95 but not in C ones. Conversely, they were accumulated in C samples of clone 76, but not in Ds+ ones. *trans*-coumaroyl-tartaric acid was accumulated in Ds+ samples of both clones but not in C samples. By contrast, rutin accumulated in C and Ds- samples of both clones but not in Ds+ samples.

**Table 2:** Accumulation of flavonoids and hydroxycinnamoyl tartaric acids in leaves.

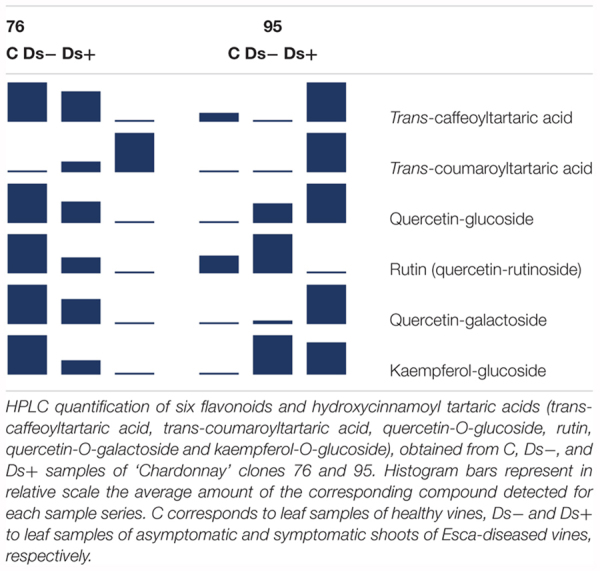

**FIGURE 7:**
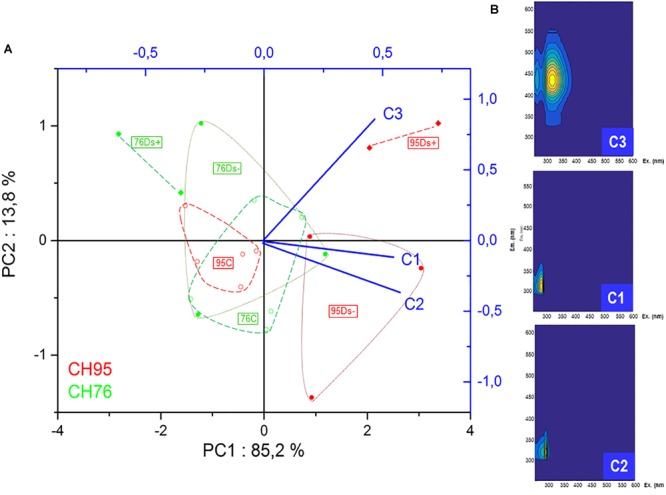
Principal component analysis and PARAFAC analysis of 3D fluorescence data. **(A)** PCA plot was built from Excitation Emission Matrix (EEM), related to the calculated PARAFAC components C1, C2, and C3 components of healthy (∘), Ds– (•) and Ds+ (♦) samples of clone 76 (in green) and clone 95 (in red). **(B)** C1, C2, and C3 components models are defined according to their emission and excitation values. C, leaf samples from healthy vines; Ds–, leaf samples from asymptomatic shoots of diseased vines; Ds+, leaf samples from symptomatic shoots of diseased vines.

3D fluorescence analysis was performed on a set of samples previously analyzed by HPLC: 5 C, 3 Ds- and 2 Ds+ from each clone. PCA analysis of the new data set was performed and the three components from PARAFAC calculation seemed to discriminate samples in a similar pattern as PCA analysis of GC-MS data obtained from all samples (Figures 3, 7). Again, Ds+ samples were perfectly isolated from C ones while distinction between Ds- and C ones was only possible for clone 95. C samples of both clones remained together, gathered with the 76Ds- ones. Interestingly, the four diseased Ds+ samples of both clones were distinctly located on the map.

## Discussion

This study was conducted in order to highlight a possible clone-dependent expression of Esca disease. It was performed in field conditions, in a plot planted the same year with the two ‘Chardonnay’ clones 76 and 95 grafted on the same rootstock. It thus allowed comparison of developmental and physiological traits, metabolome, and foliar symptom expression in the same soil and climate conditions.

Our data showed no difference in vigor among clones, a finding which is consistent with previous observations^5^. The foliar expression of Esca disease was weak and might be partly explained by the climate conditions of the experimental year. The apoplectic form of the disease is indeed generally highly expressed when a strong imbalance between water absorption and transpiration occurs, as example high soil water availability and enhanced transpiration due to high temperature and warm wind (Surico et al., 2006). In our case, pluviometry was low during the first half of the summer and delta ^13^C measurements in musts indicate a weak to moderate water stress (Gaudillere et al., 2002). The percentage of unproductive vines was also low. Vines might be too young (13 years old) to fully express the disease. If so, symptom expression will possibly increase with time (Surico et al., 2006).

Metabolite analyses were performed in leaves collected from vines without foliar symptoms (annotated C) and from both symptomatic (Ds+) and asymptomatic shoots (Ds-) of vines showing partial apoplexy symptoms. Regarding the photosynthetic pigments, no significant differences in the content of total chlorophyll and carotenoids were observed between samples. These results are not consistent with previous observations of an alteration of photosynthesis in leaves of Esca-diseased vines before apoplexy occurrence (Petit et al., 2006). It might be due to a combination of different factors such as the rootstock that was different (41B), N availability, vigor and climate conditions, and would merit to be further investigated.

For global metabolite profiling by GC-MS, PCA analysis interestingly showed that Ds+ and Ds- sample sets could be distinguished from each other in clone 95 but not in clone 76. These results suggest a clone-dependent metabolite fingerprint associated to Esca disease expression. Hierarchical clustering analysis allowed discrimination between samples of each clone. For clone 76, C leaves accumulated a higher level of secondary metabolites (kaempferol, kaempferol-3-*O*-glucoside, isoquercitrin, quercetin, a quercetin glucoside and a probable quercetin derivative, epicatechin, and gentiobiose) whereas Ds+ leaves accumulated higher amounts of *cis*-4-hydroxycinnamate and amino acids, namely threonine, leucine, isoleucine, and valine. Accumulation of amino acids have been previously reported in ‘Chardonnay’ leaves just before apoplexy (Magnin-Robert et al., 2017) (alanine), and in the xylem sap of water-stressed Esca diseased ‘Chardonnay’ vines (asparagine, isoleucine, leucine, methionine, phenylalanine, proline, tyrosine, valine) (Lima et al., 2017b). Although changes in amino acid concentration seem a common feature associated to GTDs, no specific ones can be presently considered as markers of the foliar disease expression.

Ds+ leaves of clone 95 accumulated carbohydrates as primary metabolites, together with organic acids and secondary metabolites (kaempferol, kaempferol-3-*O*-glucoside, quercetin, and putative quercetin derivative, resveratrol-glycoside, glycosyl salicylate, and gamma-tocopherol). Amino acids were therefore discriminant for Ds+ samples of clone 76, but not carbohydrates; and it was the opposite for clone 95. Such differences remain unexplained but would merit further investigation. Lima et al. (2010) also reported an impact of Esca disease on carbohydrates, amino acids and organic acids. We found higher levels of both glucose and fructose in Ds+ samples of clone 95 while they reported a decrease of these compounds in diseased ‘Alvarinho’ leaves and concluded for a mobilization of the primary metabolism toward to the secondary one. These opposite results could be due to the fact that we collected samples at the onset of symptom expression. The absence of discrimination between Ds- and Ds+ samples of each clone, and Ds- and C samples for clone 76 also goes in favor of this hypothesis. The antioxidant γ-tocopherol, also named vitamin E, was in higher amounts in Ds+ leaves of clone 95 than in C ones. In Arabidopsis, mutants (vte1 and vte4) with altered composition of α- and γ-tocopherol in chloroplasts have reduced resistance to *Botrytis cinerea* infection (Cela et al., 2018). The authors concluded that an altered tocopherol content may negatively influence the plant response to biotic stress. In Ds+ samples, the higher level of γ-tocopherol might therefore contribute to disease expression. As Esca associated fungi produce toxins (Andolfi et al., 2011; Magnin-Robert et al., 2016), it would be interesting to determine if such compounds modulate tocopherol amounts in leaves.

The accumulation of 4-hydroxycinnamate, a precursor in the biosynthesis of resveratrol (Chong et al., 2009; Adrian et al., 2012), in Ds+ leaves of clone 76, and of shikimate and resveratrol glycoside in Ds+ leaves of clone 95 suggest that plant defenses have been activated. Previous studies reported the accumulation of phytoalexins (resveratrol and derivatives such as 𝜀-viniferin) in different tissues or organs of Esca-diseased vines. As example, such compounds were found in brown-red wood (Amalfitano et al., 2000, 2011). Magnin-Robert et al. (2017) also demonstrated induction of genes involved in resveratrol biosynthesis pathway in Esca-diseased leaves prior to symptom appearance. In grapevine, phytoalexin accumulation depends on the phenological stage and seasonal pattern of Esca symptom occurrence (Calzarano et al., 2016). Gatti et al. (2014) reported a clone-dependent accumulation of stilbenes between ‘Cabernet Sauvignon’ clones but analyses were performed in berries of healthy vines. In the present study, as we did not specifically analyze resveratrol and its derivatives, we cannot compare this response with previously published results.

Interestingly, the six common discriminant metabolites (kaempferol, kaempferol-3-*O*-glucoside, quercetin, quercetin derivative, gentiobiose and an unknown U2300.3/204) pointed out between Ds+ and C samples of each clone showed opposite variation: they accumulated in higher amounts in Ds+ leaves of clone 95 and in lower amounts in Ds+ leaves of clone 76. This observation suggests that the amounts of these compounds in leaves are clone and Esca-disease-dependent. However, we cannot correlate their accumulation in leaves with symptom expression of Esca disease. Gentiobiose (6-*O*-ß-D-glucopyranosyl-D-glucose) is an up-regulator of the synthesis of glutathione and an activator in the ascorbate/glutathione cycle (Takahashi et al., 2014). Interestingly, the regulation of glutathione metabolism was previously reported in leaves of Esca-affected vines (Valtaud et al., 2009; Letousey et al., 2010; Magnin-Robert et al., 2017). Four other discriminant compounds are phenolics. Accumulation of such compounds in plant-microbe interactions is well documented. As example, Koskimäki et al. (2009) have reported that Bilberry infection by the fungal endophyte *Paraphaeosphaeria* sp. and by the pathogen *Botrytis cinerea* induced the accumulation of quercetin-3-*O*-glucoside and two other glucosides. Similarly, Rusjan et al. (2012) observed modifications of phenolics in ‘Chardonnay’ in response to Bois Noir phytoplasma. They found a lower content of quercetin-3-*O*-glucoside in ripening berries of healthy vines, compared to berries from (a)symptomatic infected vines. More recently, Lima et al. (2017a) performed an experiment similar to the present one by HPLC analysis of phenolics in ‘Alvarinho’ vines and managed to discriminate leaves of “healthy” vines from symptomatic and asymptomatic leaves of Esca-affected ones. They indeed observed an accumulation of *trans*-caffeoyltartaric acid, *trans*-coumaroyl-tartaric acid, quercetin-3-*O*-glucoside, quercetin-3-*O*-galactoside, kaempferol-3-*O*-glucoside, and myricetin in symptomatic leaves, and, to a lesser extent, in asymptomatic leaves of Esca-diseased vines, compared to leaves of healthy vines. In the present study, the metabolomic analysis mainly targeted primary metabolites, and only few secondary metabolites were therefore identified. Quercetin, kaempferol, and some of their derivatives were nevertheless recurrently highlighted as discriminant compounds of C versus Ds+ samples for both clones.

Lima et al. (2017a) previously reported a correlation between the accumulation of flavonoids and also hydroxycinnamoyl tartaric acids and Esca-disease expression, with lower amounts in apparently healthy leaves. HPLC analysis of most of the relevant metabolites reported by these authors (i.e., *trans*-caffeoyltartaric acid, *trans*-coumaroyl-tartaric acid, quercetin-3-*O*-glucoside, quercetin-3-*O*-galactoside, and kaempferol-3-*O*-glucoside) was therefore performed for comparison. The clone-dependent accumulation of these compounds could be confirmed. As reported by Lima et al. (2017a), *trans*-caffeoyltartaric acid, quercetin-*O*-glucoside, quercetin-*O*-galactoside, and kaempferol-*O*-glucoside were accumulated in Ds+ samples of clone 95 but not in C ones. Conversely, they were accumulated in C samples of clone 76, but not in Ds+ ones. Hence, it looks like that in response to Esca disease, ‘Chardonnay’ clone 95 behaves in a similar manner as cv. ‘Alvarinho’ while ‘Chardonnay’ clone 76 responds differently, in an opposite manner. Altogether, this study points out the involvement of *trans*-caffeoyltartaric acid, *trans*-coumaroyltartaric acid, quercetin-*O*-glucoside, quercetin-*O*-galactoside, kaempferol-*O*-glucoside and gentiobiose in Esca disease expression in leaves. It would be interesting to compare their profile of accumulation in leaves of healthy and Esca-diseased vines of a larger number of ‘Chardonnay’ clones, and also in different varieties. Additionally, their accumulation would merit to be monitored before and at different time points of foliar symptom expression.

Some leaf samples were further used to check if 3D fluorescence analysis could also demonstrate significant differences in disease expression between studied clones. The three components from PARAFAC calculation allowed sample discrimination in a similar pattern as PCA analysis of GC-MS data obtained from all analyzed samples. Although the fluorescent metabolites involved in the 3 components of the PARAFAC map remain at this point non-identified, this method seems suitable to detect the impact of Esca-disease in leaves and confirms its clone-dependent expression. This is the first time, to our knowledge, that this method is applied to the study of plant-pathogen interaction. Ds+ samples were clearly distinguished in terms of elevated *F*_max_ values for PARAFAC component C3 that were also richer in plant metabolites belonging to the family of glycosylated flavonols and cinnamic acid esters of tartaric acid, as previously proposed in wine matrices (Roullier-Gall et al., 2017). It would be interesting to extend the analysis to other clones and varieties and to assess its potential to easily discriminate healthy and diseased samples in other plant/pathogens interactions and at different stages of infection. Moreover, 3D fluorescence analysis could be explored for its potential as precocious detection of diseases or abiotic stresses of plants in field conditions.

## Author Contributions

FF, GuM, and MA designed the work. CG performed the sampling. LJ contributed to sample preparation and performed the leaf pigment analysis. GC and GrM performed the GC-MS analysis. JN performed the HPLC analysis. CC and CL-G performed the 3D-fluorescence analyses. CL-G and FM performed the data analysis. CC, CL-G, FF, FM, GC, GuM, JN, MA, and ST contributed to data interpretation. CL-G, FM, and MA drafted the manuscript. All authors reviewed, edited, and approved the manuscript.

## Conflict of Interest Statement

The authors declare that the research was conducted in the absence of any commercial or financial relationships that could be construed as a potential conflict of interest.

## Footnotes

1 http://chemdata.nist.gov/mass-spc/amdis/

2 http://gmd.mpimp-golm.mpg.de/

3 http://www.perseus-framework.org

4 http://www.rstudio.com/

5 http://plantgrape.plantnet-project.org/fr/cepage/Chardonnay#76

## Supplementary Material

FIGURE S1 Climate data. Temperature and rain data were provided by a meteorological station (Cimmel Enerco 404) located close to the plot, and representative for the climate conditions of the experimental plot. Mean temperatures and rainfalls were calculated monthly for 2015 (gray spots and bars, respectively) and over a 10-year period (2005–2015) (black spots and bars, respectively).

FIGURE S2 Heatmap of metabolites intensity following hierarchical clustering. Values resulting from quantification of the 177 compounds detected in leaf extracts of ‘Chardonnay’ clones 76 and 95 were loaded onto Perseus software (version 1.5.1.6, http://www.perseus-framework.org), which performed the z-scoring. ANOVA multivariate analysis was applied with a *P* < 0.05. From this, 75 significant metabolites were retained and clustered with a Pearson correlation. Samples are annotated and colored on the top of the map, and the 75 significant compounds are located on the left hand side. Gradient between green and red color means less to over accumulation of the corresponding compound. C corresponds to leaf samples of healthy vines, Ds+ and Ds- to leaf samples of symptomatic and asymptomatic shoots of Esca diseased vines (five biological replicates).

TABLE S1 GC-MS data matrix. Annotated matrix of the metabolites analyzed by GC-MS in clones 76 and 95 leaf samples.

TABLE S2 Temperature data. Values ± standard deviation of the average monthly temperature for each month over 10 years (2005–2014) and for 2015 with minimum and maximum temperatures (°C).

TABLE S3 Leaf pigment analysis. Levels of leaf pigments in samples of ‘Chardonnay’ clones 76 and 95. Lowercase letters (a and b) indicate significant difference between modalities determined by Kruskal–Wallis test followed by Dunn’s sum-rank pairwise *post hoc* test with Holm correction (*P* < 0.05). C corresponds to leaf samples of healthy vines, Ds+ and Ds- to leaf samples of symptomatic and asymptomatic shoots of Esca diseased vines. Chl, chlorophyll; Car, carotenoids; ChlTot, total chlorophyll.
