# Supplementary figures and images for: Clone-Dependent Expression of Esca Disease Revealed by Leaf Metabolite Analysis
Source: Front Plant Sci. 2019 Jan 9;9:1960. doi: 10.3389/fpls.2018.01960 (PMC6333860; doi:10.3389/fpls.2018.01960)

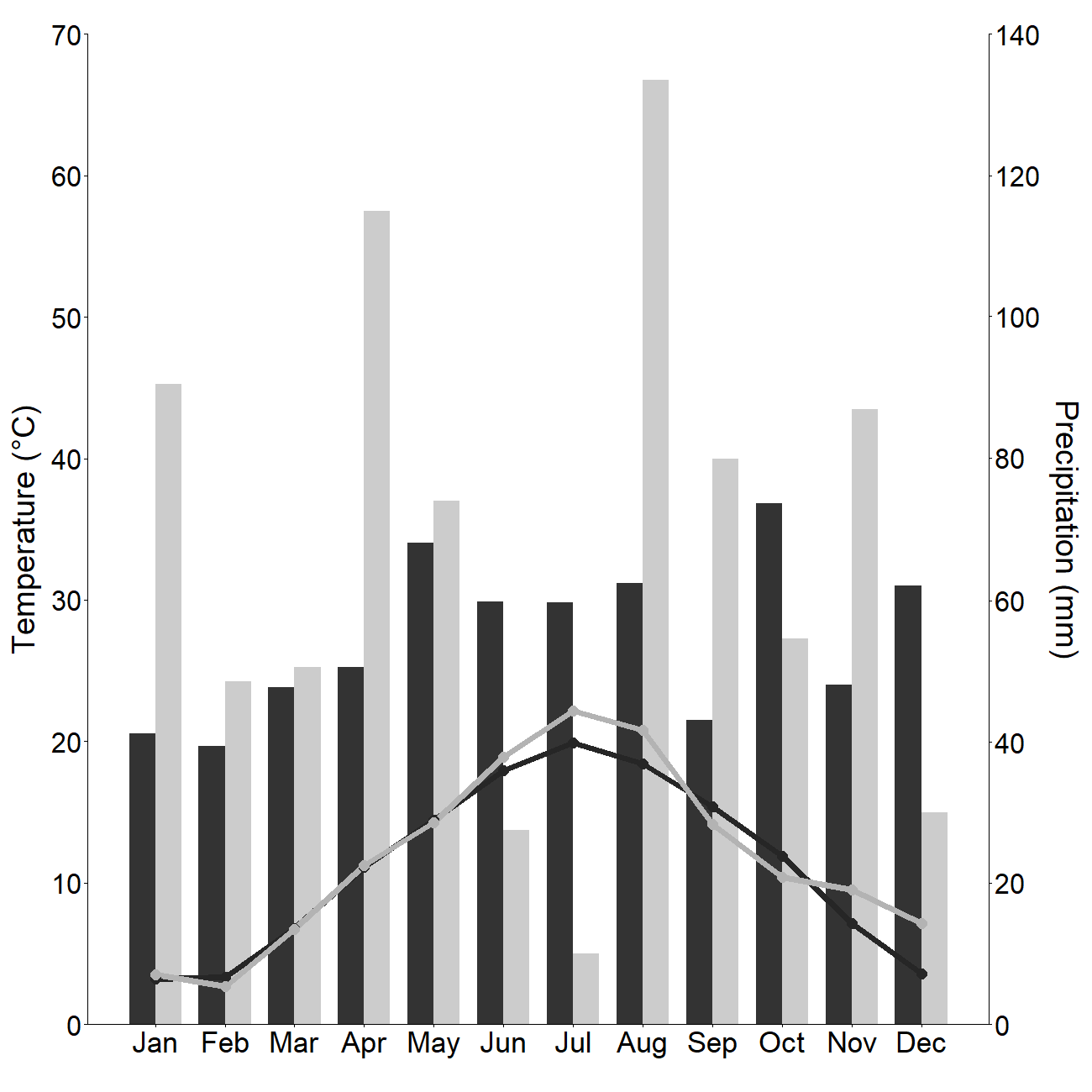

Supplement: FIGURE S1 — Climate data. Temperature and rain data were provided by a meteorological station (Cimmel Enerco 404) located close to the plot, and representative for the climate conditions of the experimental plot. Mean temperatures and rainfalls were calculated monthly for 2015 (gray spots and bars, respectively) and over a 10-year period (2005–2015) (black spots and bars, respectively). [file Image_1.TIF]

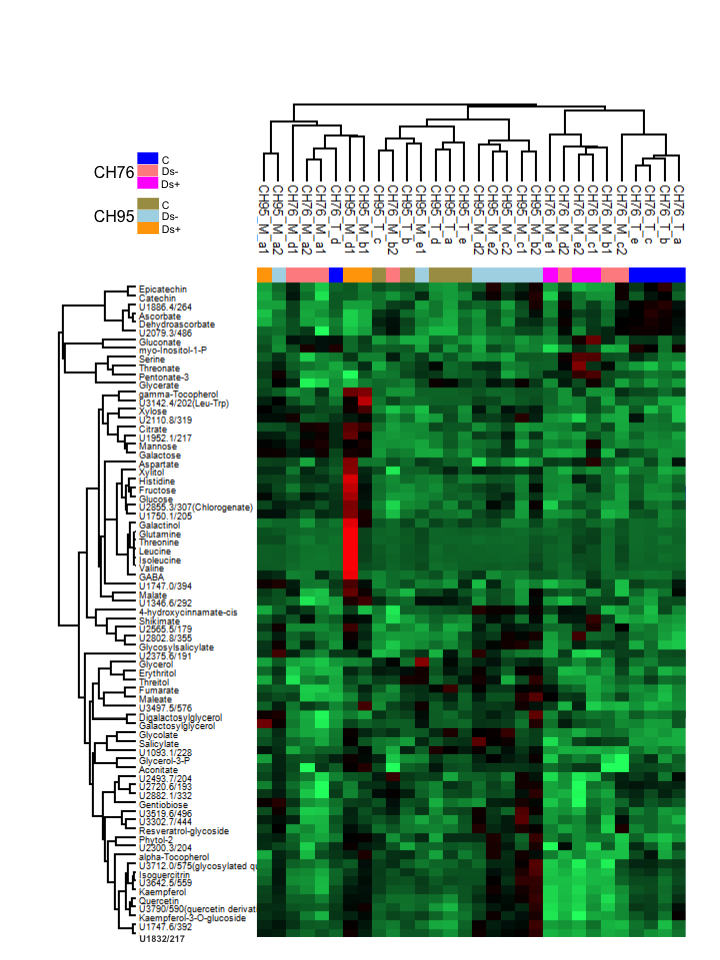

Supplement: FIGURE S2 — Heatmap of metabolites intensity following hierarchical clustering. Values resulting from quantification of the 177 compounds detected in leaf extracts of ‘Chardonnay’ clones 76 and 95 were loaded onto Perseus software (version 1.5.1.6, http://www.perseus-framework.org), which performed the z-scoring. ANOVA multivariate analysis was applied with a P < 0.05. From this, 75 significant metabolites were retained and clustered with a Pearson correlation. Samples are annotated and colored on the top of the map, and the 75 significant compounds are located on the left hand side. Gradient between green and red color means less to over accumulation of the corresponding compound. C corresponds to leaf samples of healthy vines, Ds+ and Ds- to leaf samples of symptomatic and asymptomatic shoots of Esca diseased vines (five biological replicates). [file Image_2.tiff]
